# Interferon-Induced *Ifit2/ISG54* Protects Mice from Lethal VSV Neuropathogenesis

**DOI:** 10.1371/journal.ppat.1002712

**Published:** 2012-05-17

**Authors:** Volker Fensterl, Jaime L. Wetzel, Srividya Ramachandran, Tomoaki Ogino, Stephen A. Stohlman, Cornelia C. Bergmann, Michael S. Diamond, Herbert W. Virgin, Ganes C. Sen

**Affiliations:** 1 Department of Molecular Genetics, Lerner Research Institute, Cleveland Clinic, Cleveland, Ohio, United States of America; 2 Department of Neurosciences, Lerner Research Institute, Cleveland Clinic, Cleveland, Ohio, United States of America; 3 Departments of Medicine, Molecular Microbiology, and Pathology & Immunology, Washington University School of Medicine, St. Louis, Missouri, United States of America; Vaccine & Gene Therapy Institute of Florida, United States of America

## Abstract

Interferon protects mice from vesicular stomatitis virus (VSV) infection and pathogenesis; however, it is not known which of the numerous interferon-stimulated genes (ISG) mediate the antiviral effect. A prominent family of ISGs is the interferon-induced with tetratricopeptide repeats (*Ifit*) genes comprising three members in mice, *Ifit1/ISG56*, *Ifit2/ISG54* and *Ifit3/ISG49*. Intranasal infection with a low dose of VSV is not lethal to wild-type mice and all three *Ifit* genes are induced in the central nervous system of the infected mice. We tested their potential contributions to the observed protection of wild-type mice from VSV pathogenesis, by taking advantage of the newly generated knockout mice lacking either *Ifit2* or *Ifit1*. We observed that in *Ifit2* knockout (*Ifit2* ^−/−^) mice, intranasal VSV infection was uniformly lethal and death was preceded by neurological signs, such as ataxia and hind limb paralysis. In contrast, wild-type and *Ifit1* ^−/−^ mice were highly protected and survived without developing such disease. However, when VSV was injected intracranially, virus replication and survival were not significantly different between wild-type and *Ifit2^−/−^* mice. When administered intranasally, VSV entered the central nervous system through the olfactory bulbs, where it replicated equivalently in wild-type and *Ifit2* ^−/−^ mice and induced interferon-β. However, as the infection spread to other regions of the brain, VSV titers rose several hundred folds higher in *Ifit2* ^−/−^ mice as compared to wild-type mice. This was not caused by a broadened cell tropism in the brains of *Ifit2* ^−/−^ mice, where VSV still replicated selectively in neurons. Surprisingly, this advantage for VSV replication in the brains of *Ifit2^−/−^* mice was not observed in other organs, such as lung and liver. Pathogenesis by another neurotropic RNA virus, encephalomyocarditis virus, was not enhanced in the brains of *Ifit2* ^−/−^ mice. Our study provides a clear demonstration of tissue-, virus- and ISG-specific antiviral action of interferon.

## Author Summary

In mammals, the first line of defense against virus infection is the interferon system. Viruses induce synthesis of interferon in the infected cells and its secretion to circulation. Interferon acts upon the as yet uninfected cells and protects them from oncoming infection by inducing the synthesis of hundreds of new proteins, many of which interfere with virus replication. Vesicular stomatitis virus (VSV), a virus similar to rabies virus, is very sensitive to interferon but it is not known which interferon-induced protein inhibits its replication. Here, we have identified a single interferon-induced protein as the protector of mice from death by VSV infection. Knocking out the gene encoding this protein, Ifit2, made mice very vulnerable to neuropathogenesis caused by VSV infection; a related protein, Ifit1, did not share this property. Moreover, Ifit2 failed to protect mice from another neurotropic virus, encephalomyocarditis virus, nor was it necessary for protecting organs other than brain from infection by VSV. Our observation that a single IFN-induced protein protects a specific organ from infection by a specific virus revealed an unexpected degree of specificity of the antiviral action of IFN.

## Introduction

Virus infection of mammals induces the synthesis of type I interferons (IFN), which, in turn, inhibit virus replication. The high susceptibility of type I IFN receptor knockout (*IFNAR^−/−^*) mice to infection by a variety of viruses [1]–[3] provides strong evidence for the major role of the IFN system in protecting from viral pathogenesis. In these mice, although IFN is induced by virus infection, it cannot act on target cells. Similarly, in genetically altered mice that are defective in IFN production due to the absence of specific pathogen-associated pattern recognition receptors, signaling proteins or specific transcription factors, viral pathogenesis is enhanced [4]–[6]. Although the critical importance of the IFN system in regulating viral pathogenesis is now well established, in many cases it is still unclear how IFN inhibits the replication and spread of a specific virus *in vivo*. In this context, activation of different components of the immune system plays a major role in controlling viral diseases that are relatively slow to develop [7]–[9]. In contrast, in acute infection by viruses that cause severe pathogenesis and death within a few days after infection, protection is primarily provided by the intrinsic antiviral actions of IFN-induced proteins encoded by the hundreds of IFN-stimulated genes (ISGs) [10]–[12], several of which often contribute to the overall effect of IFN against a given virus. Our knowledge of the antiviral and the biochemical properties of individual ISG products is mostly limited to a few intensively studied examples such as PKR, OAS/RNase L or Mx [13]. However, recent systematic investigation of the antiviral functions of the entire family of ISGs has started producing exciting new information [14].

In the above context, we have been investigating the biochemical and biological functions of the members of the *Ifit* family of ISGs, which are very strongly induced by IFN. There are three members of this family of genes in mice: *Ifit1/ISG56*, *Ifit2/ISG54* and *Ifit3/ISG49*; all of the encoded proteins contain multiple tetratricopeptide repeats (TPR), which mediate protein-protein and protein-RNA interactions [15]. *In vitro*, P56 and P54, the products of *Ifit1* and *Ifit2*, respectively, bind to the translation initiation factor eIF3 and inhibit protein synthesis [16]. The third member, P49, the product of *Ifit3*, does not share this property [17]. Recently, it has been reported that Ifit proteins form a multi-protein complex that can bind to the triphosphorylated 5′ end of RNAs, an RNA-species produced during the replication of some, but not all, viruses [18]. *In vivo*, these genes are strongly induced in brains of mice infected with West Nile virus (WNV) or Lymphocytic choriomeningitis virus (LCMV); surprisingly, different *Ifit* genes are differentially induced in different regions of the brain, suggesting non-redundant functions [19]. To further explore the antiviral properties of the Ifit proteins, we generated *Ifit1* knockout (*Ifit1^−/−^*) mice and challenged them with different viruses. We observed that *Ifit1^−/−^* mice were particularly susceptible to a WNV mutant that is defective in its mRNA cap 2′-*O* methylation; the mutant virus killed *Ifit1^−/−^* mice but not the wild-type (wt) mice [20].

Here, we report on the antiviral properties of the newly generated *Ifit2^−/−^* mice; these mice, but not *Ifit1^−/−^* mice, were highly susceptible to neuropathogenesis after intranasal infection with vesicular stomatitis virus (VSV), a negative sense, single-stranded RNA rhabdovirus. VSV replication is highly sensitive to the inhibitory action of IFN and is routinely used to assay the antiviral activity of IFN *in vitro* [21]. As expected, *IFNAR^−/−^* mice are highly susceptible to VSV pathogenesis and the same is true for mice that specifically lack expression of IFNAR on the cells of their central nervous system (CNS) [1]. In spite of these observations, little is known about how IFN inhibits VSV replication *in vivo*. Our new results indicate that in the brain, but not in other organs, *Ifit2* is a major mediator of IFN's protective effect against VSV. In contrast, *Ifit2* could not protect mice from neuropathogenesis caused by encephalomyocarditis virus (EMCV), a picornavirus. Thus, we have uncovered a virus-specific, tissue-specific and ISG-specific antiviral effect of the IFN system.

## Results

### Generation of *Ifit2/ISG54* and *Ifit1/ISG56* knockout mice

**Figure 1:**
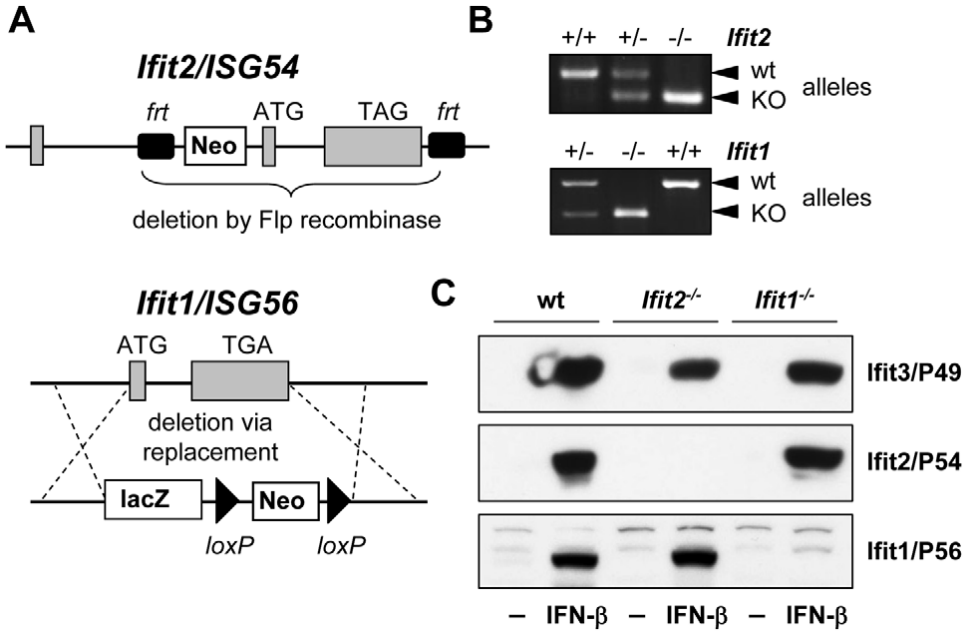
Generation of *Ifit2/ISG54* and *Ifit1/ISG56* knockout mice. **A**, gene targeting strategy for genomic deletion of complete protein-encoding regions of *Ifit2 = ISG54* or *Ifit1 = ISG56* in embryonic stem cells; TAG/TGA, stop codons; grey boxes = exons. **B**, genotyping of deficiency for *Ifit2* or *Ifit1* by PCR on mouse tail DNA. **C**, IFN-β-induced protein expression of Ifit2/P54, Ifit1/P56 and Ifit3/P49 in *Ifit2* ^−/−^ or *Ifit1^−/−^* primary MEFs.

*Ifit2* gene knockout (*Ifit2* ^−/−^) mice were generated by deleting the entire protein-encoding region of the gene, which was achieved by flanking exons 2 and 3 with *frt* recombinase sites in C57BL/6 embryonic stem cells and excising the flanked region with Flp recombinase (Figure 1A). *Ifit2* ^−/−^ mice were bred to homozygosity (Figure 1B), and deficiency for induced expression of Ifit2 protein was confirmed in lysates of IFN-β-treated primary murine embryonic fibroblasts (MEF) (Figure 1C). Mice deficient for *Ifit1* (*Ifit1^−^* ^/−^) were derived from C57BL/6 embryonic stem cells lacking the entire *Ifit1* coding region (Figure 1A). Genotypic homozygosity of the *Ifit1^−^* ^/−^ mice and deficiency for Ifit1 protein induction were confirmed (Figure 1B and 1C). Both knockout mouse lines were healthy and fertile. Moreover, deletion of one gene within the *Ifit* locus did not alter the pattern of induction of other adjacent gene family members, as compared to wild-type (wt) mice (Figure 1C).

### *Ifit2* protects mice from lethal intranasal VSV infection

**Figure 2:**
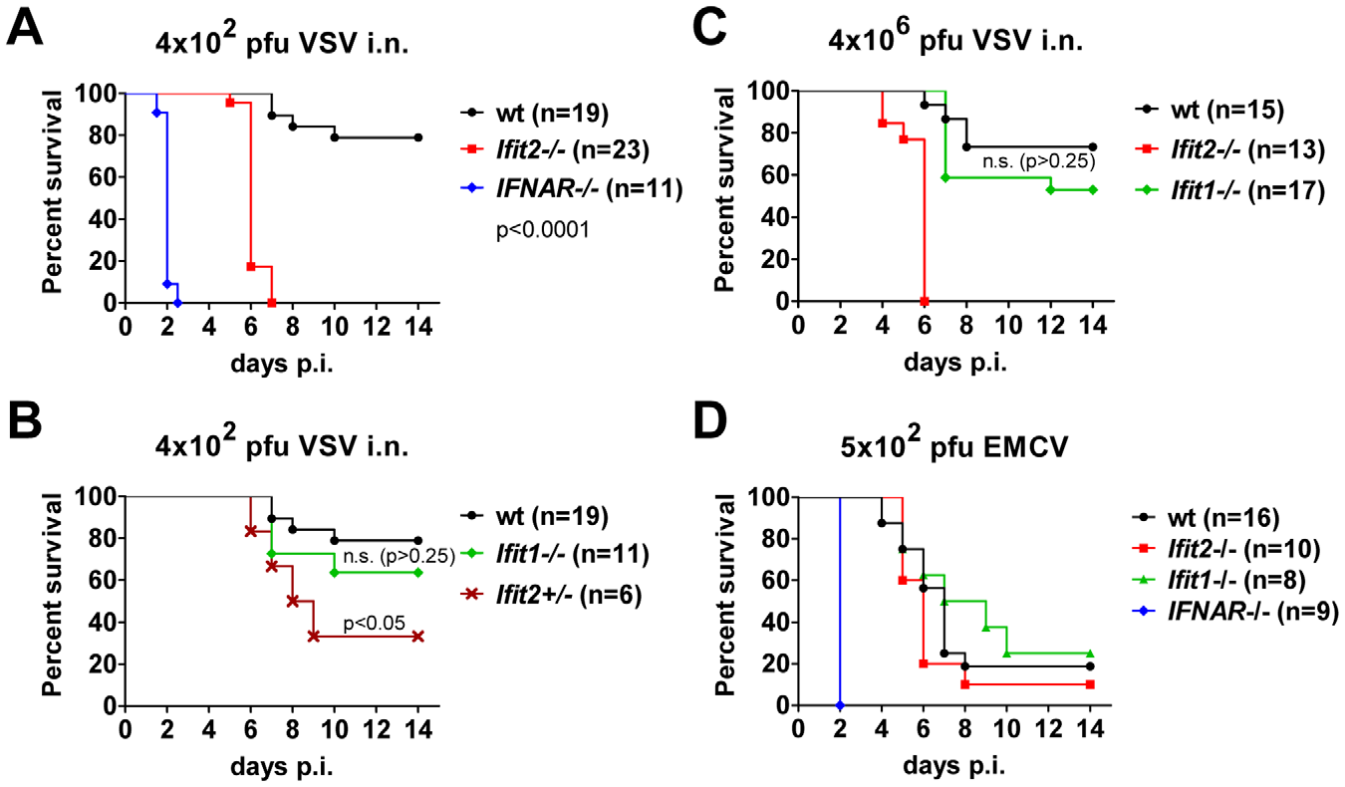
*Ifit2* protects mice from lethal intranasal VSV infection. **A**, survival of *Ifit2^−/−^*, *IFNAR* ^−/−^ and wt mice after intranasal infection with 4×10^2^ pfu of VSV Indiana. **B**, survival of *Ifit1^−/−^* and *Ifit2*-heterozygous (*Ifit2^+/−^*) mice after infection with 4×10^2^ pfu of VSV; experiments in A and B shared wt mice (n = number of animals used). **C**, survival of *Ifit2^−/−^*, *Ifit1^−/−^* and wt mice after intranasal infection with a higher dose of VSV (4×10^6^ pfu). **D**, survival of *Ifit2^−/−^*, *Ifit1^−/−^*, *IFNAR* ^−/−^ and wt mice after infection with 5×10^2^ pfu of EMCV. In A–D, data are cumulative from at least two independent experiments (exceptions: Figure 2B, *Ifit2* ^+/−^ mice and Figure 2D, *Ifit1* ^−/−^ mice infected in a single experiment). Statistical significance of survival differences relative to wt mice is indicated by p-values; n.s., not significant; i.n., intranasal.

To determine the impact of *Ifit2* on the outcome of viral infections *in vivo*, we compared susceptibilities of *Ifit2^−/−^* and wt mice to VSV infection, using *IFNAR* ^−/−^ mice as positive controls of enhanced susceptibility. Virus was administered at a low dose [4×10^2^ plaque forming units (pfu)], intranasally, reflecting a natural route of infection for VSV [22]. As seen previously, 100% of *IFNAR* ^−/−^ mice rapidly succumbed to VSV infection within 2 days (Figure 2A, and [1]), after suffering symptoms of lethargy. On the other hand, 79% of wt mice survived, the remaining 21% succumbed to VSV, and this occurred later, at 7–10 days post infection (d.p.i.). In contrast, 100% of *Ifit2^−/−^* mice died by 7 d.p.i. (Figure 2A), with most succumbing by 6 d.p.i.; thus, we observed uniform and more rapidly occurring death of *Ifit2^−/−^* compared to wt mice after VSV infection. Within 24 h before death, both wt and *Ifit2^−/−^* mice developed neurological signs including ataxia, hind limb paralysis, and hyper-excitability. *Ifit2* ^+/−^ mice displayed an intermediate survival curve, demonstrating a gene dosage effect (Figure 2B). Next, the role of a related gene, *Ifit1*, in VSV pathogenesis was evaluated by infecting *Ifit1* ^−/−^ mice. Unlike the results observed with *Ifit2^−/−^* mice, no statistically significant increase in mortality was observed in *Ifit1^−/−^* mice (Figure 2B, 21% death for wt versus 36% for *Ifit1* ^−/−^, respectively; p>0.25). Consistent with this, survival kinetics of *Ifit1^−/−^* and wt mice were similar. Increasing the virus dose by 10,000-fold (to 4×10^6^ pfu) did not appreciably change the survival curves of wt, *Ifit1^−/−^*, or *Ifit2^−/−^* mice (Figure 2C). These results demonstrate functional differences between the two closely related proteins encoded by *Ifit1* and *Ifit2*. The virus-specificity of the antiviral action of *Ifit2* was evaluated by infecting *Ifit2^−/−^* mice with EMCV, an unrelated neurovirulent positive-strand RNA virus of the picornavirus family (Figure 2D). *IFNAR* ^−/−^ mice were highly susceptible to EMCV infection with all mice succumbing within 2 d.p.i.; in contrast, wt mice died with a slower kinetics and at a rate of only 80%. Notably, *Ifit2^−/−^* mice behaved similarly to the wt mice, without enhanced or accelerated mortality (Figure 2D). The same conclusion was true for a lower dose of EMCV (Figure S1). The survival pattern of EMCV-infected *Ifit1* ^−/−^ mice also was similar to that of the wt mice (Figure 2D). Mice of all genotypes either succumbed after developing neurological symptoms, mainly hind limb paralysis, or survived without symptoms. These results demonstrate that the antiviral action of *Ifit2* is both virus- and *Ifit*-specific.

### *Ifit2* does not inhibit VSV entry and replication in olfactory bulbs

**Figure 3:**
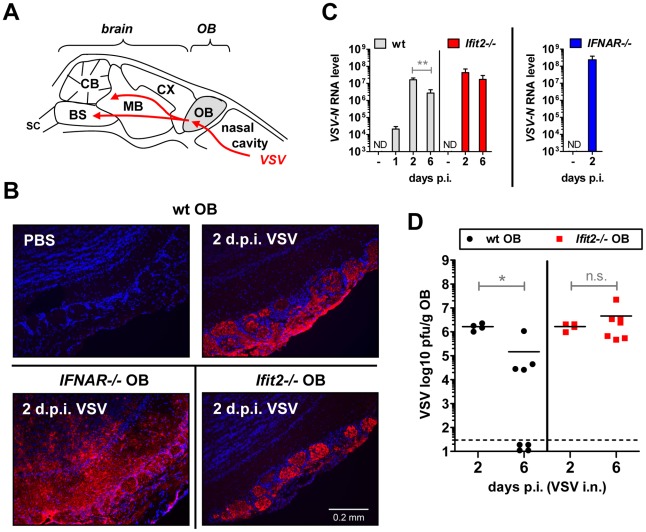
*Ifit2* does not inhibit VSV entry and replication in olfactory bulbs. **A**, schematic entry route of VSV into the central nervous system of wt mice after intranasal infection, and VSV spread within brain, as reported in the literature. OB, olfactory bulbs; CX, cortex; MB, midbrain; CB, cerebellum; BS, brain stem; SC, spinal cord. **B**, VSV P protein in OB of VSV-infected wt, *Ifit2* ^−/−^ and *IFNAR* ^−/−^ mice at 2 d.p.i., detected by immunohistofluorescence. **C**, VSV RNA levels in OB of uninfected or VSV-infected wt, *Ifit2* ^−/−^ and *IFNAR* ^−/−^ mice at 1, 2 or 6 d.p.i., plotted as mean+SD on log scale; ND, none detected. **D**, infectious VSV titers in wt and *Ifit2* ^−/−^ OB at 2 and 6 d.p.i.; plotted as pfu/g with mean on log scale; dashed line depicts threshold of detection. In C and D, n = 4–8 mice per infected group accumulated from three independent experiments; in B, n = 2 mice from two independent experiments. All infections were 4×10^2^ pfu of VSV administered intranasally. Asterisks indicate statistical significance: ** p = 0.006, * p<0.05; n.s.: not significant.

The uniform penetrance of neuropathogenesis and lethality of VSV-infected *Ifit2^−/−^* mice, even at a low virus dose, prompted us to examine viral spread along its route from the nasal cavity into the CNS (Figure 3A). After intranasal administration, VSV infects the nasal epithelia including olfactory sensor neurons, which project to the outer layer of the olfactory bulbs (OB) [23]. This represents the entry step into the CNS, which we examined by immunostaining of OB sections. In wt mice, VSV P protein was detected exclusively within the glomeruli of the OB at 2 d.p.i. (Figure 3B, *upper right panel* and [1]), whereas in *IFNAR* ^−/−^ mice, VSV antigen had spread into deeper layers of the OB (Figure 3B, *lower left panel*). In *Ifit2* ^−/−^ mice OB, viral antigen was restricted to the glomeruli, as seen in wt mice (Figure 3B, *lower right panel*). This similar pattern of viral antigen expression between wt and *Ifit2* ^−/−^ mice was reflected in the equivalent levels of viral RNA in OB at 2 d.p.i. (Figure 3C). In contrast, ∼10 times more VSV RNA was present in OB of *IFNAR* ^−/−^ mice (Figure 3C, *right panel*, p<0.05). A comparison of the infectious viral burden between wt and *Ifit2* ^−/−^ mice in the OB confirmed these findings: at 2 d.p.i., ∼10^6^ pfu/g of VSV was present in both wt and *Ifit2* ^−/−^ mice (Figure 3D, *p = 1.0*). However, later in the course of infection, by day 6, viral OB titers in *Ifit2* ^−/−^ mice were not significantly changed, whereas in wt mice average titers of infectious VSV as well as viral RNA levels had decreased by ∼10-fold (Figure 3C and D, both p<0.05). These results suggest that VSV initially enters and replicates with similar efficiency in both wt and *Ifit2* ^−/−^ OB before spreading into the rest of the brain.

### *Ifit2* suppresses replication of VSV in the brain after intranasal infection

**Figure 4:**
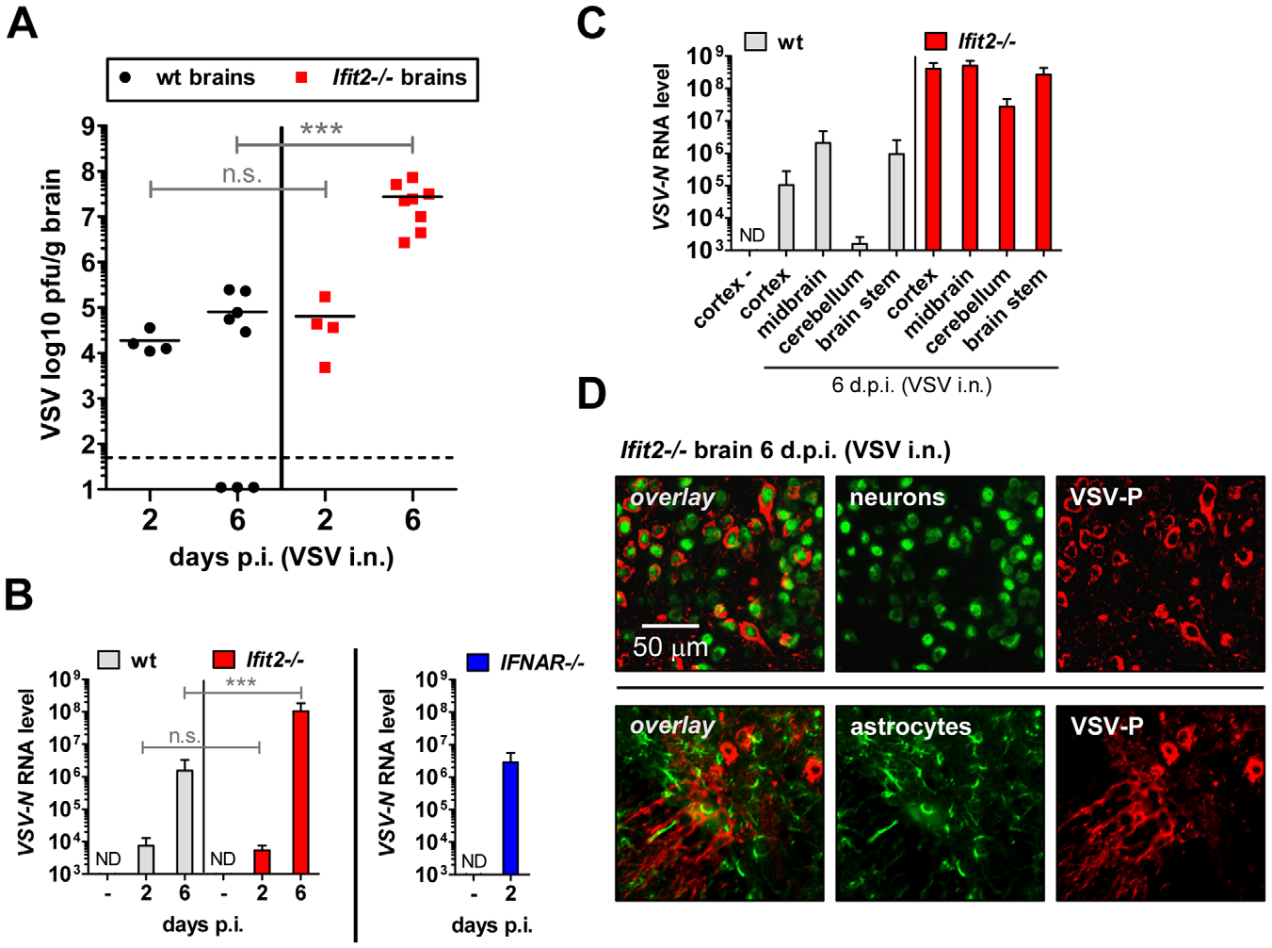
*Ifit2* suppresses VSV replication in the brain after intranasal infection. **A**, infectious VSV titers in wt and *Ifit2* ^−/−^ brains at 2 and 6 days after intranasal infection, plotted as pfu/g with mean on log scale; dashed line depicts threshold of detection. **B**, VSV RNA levels in brains of uninfected or VSV-infected wt, *Ifit2* ^−/−^ and *IFNAR* ^−/−^ mice at 2 or 6 d.p.i., plotted as mean+SD on log scale. **C**, VSV RNA levels in different regions of the brains of uninfected or VSV-infected wt and *Ifit2* ^−/−^ mice at 6 d.p.i., plotted as mean+SD on log scale. **D**, VSV P protein in midbrain neurons of *Ifit2* ^−/−^ mice at 6 d.p.i.; detection by immunohistofluorescence-labeling of VSV-P (red) and neuron (NeuN) or astrocyte (GFAP) markers (green); in A and B: n = 4–8 mice per infected group accumulated from three independent experiments; in C: n = 4 mice per infected group; in D: n = 2 mice per infected group; all infections in A–D were intranasal with 4×10^2^ pfu of VSV. ND, none detected. Brains in A and B were separated from OBs assayed in Figure 3D and 3C, respectively. Asterisks indicate statistical significance: *** p≤0.0009; n.s.: not significant.

The efficiency of VSV replication in the brain, excluding the OB, was examined by quantifying infectious VSV as well as viral RNA. Early after infection, at 2 d.p.i., virus titers in brains were low (∼10^4^ to 10^5^ pfu/g) and roughly equivalent in wt and *Ifit2* ^−/−^ mice (Figure 4A, p>0.25). Similarly, viral RNA levels at the same time were low and comparable between wt and *Ifit2* ^−/−^ (Figure 4B, p>0.25). However, at the same time, levels of VSV RNA (380-fold, p<0.05) were much higher in the brains of *IFNAR* ^−/−^ mice (Figure 4B, right panel). Later in the course of infection (6 d.p.i.), brains of wt mice accumulated only ∼5-fold more infectious VSV, with occasional clearance of the virus. In contrast, we detected markedly higher VSV titers in the brains of *Ifit2* ^−/−^ mice (∼350-fold higher compared to wt mice, p = 0.0009), reaching ∼10^8^ pfu/g (Figure 4A); the high virus load likely caused the pronounced lethality. Differences in viral RNA levels in brains of wt and *Ifit2* ^−/−^ mice at 6 d.p.i. correlated well with levels of infectious VSV (Figure 4B). To determine whether *Ifit2* selectively restricts replication of VSV in particular regions of the brain, we measured viral RNA levels in cortex, midbrain, cerebellum and brain stem at 6 d.p.i. In wt mice, VSV RNA was present prominently in the cortex, midbrain and brainstem, but not in the cerebellum (Figure 4C), which is consistent with published results [24]. However, in *Ifit2^−^* ^/−^ mice, viral RNA was 200-fold or more (p<0.05) abundant in all regions of the brain examined, including the cerebellum. The increase of VSV replication in *Ifit2* ^−/−^ brains was not due to a broadened cell tropism of the virus; immunostaining for viral P protein showed exclusive localization to neurons and not other cell types, such as astrocytes (Figure 4D). From the above observations, we conclude that after intranasal infection by VSV, *Ifit2* protects mice from neuropathogenesis by suppressing replication or spread of the virus in brain neurons.

### *Ifit2* and *Ifit1* are induced in VSV-infected regions of OB and brain

**Figure 5:**
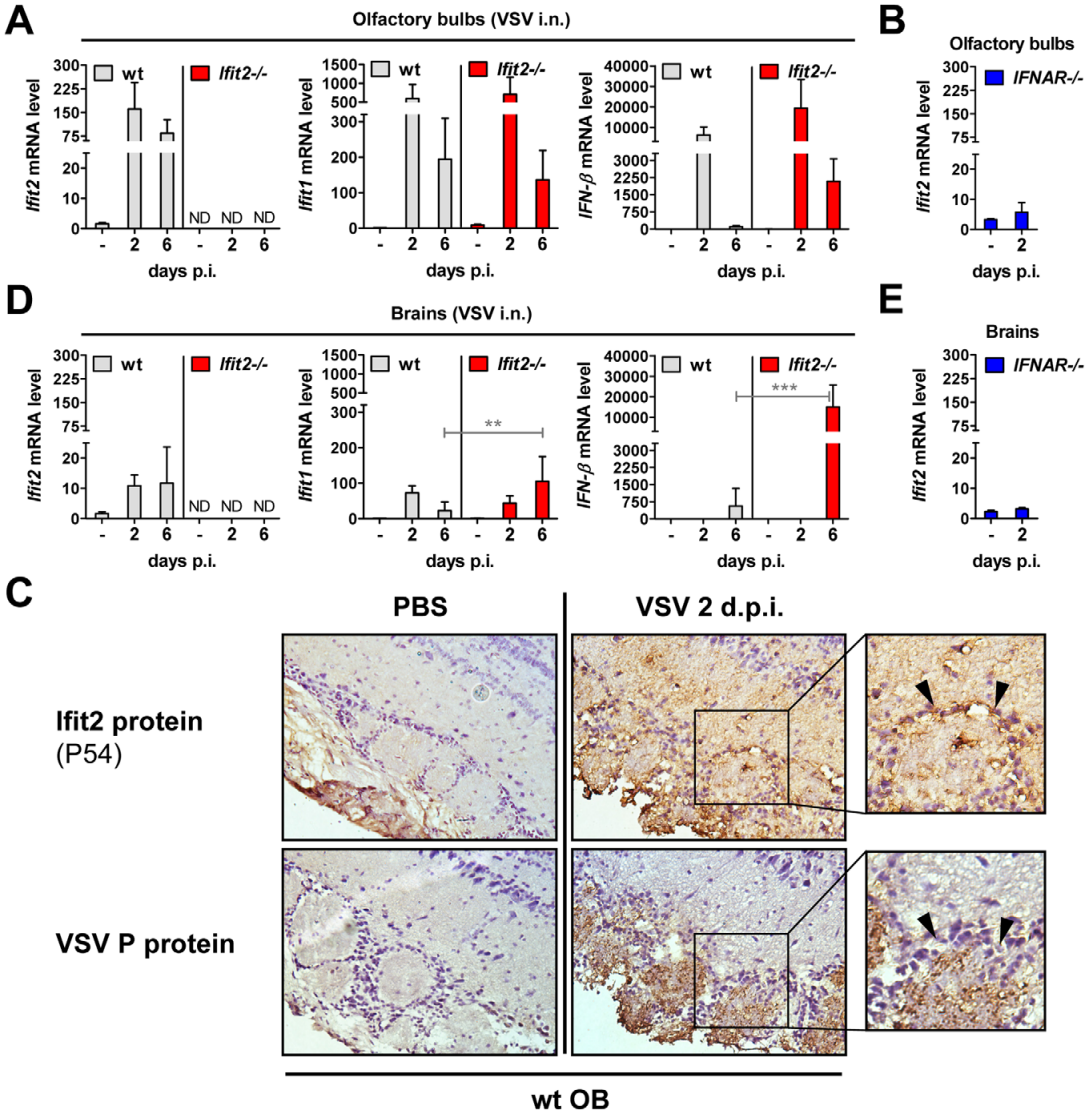
*Ifit2* and *Ifit1* are induced in VSV-infected regions of OB and brain. **A**/**B**, **D/E**, *Ifit2*, *Ifit1* and *IFN-β* mRNA levels in OB (A, B) and separated brains (D, E) of uninfected or intranasally VSV-infected wt, *Ifit2* ^−/−^ and *IFNAR* ^−/−^ mice at 2 or 6 d.p.i., plotted as mean+SD. The same OB and brains were also assayed in Figure 3C and 4B for VSV RNA levels. In A/B/D/E: n = 4–8 mice per infected group accumulated from three independent experiments; ND, none detected. **C**, Ifit2 ( = P54) protein in wt OB sections, uninfected or 2 d.p.i., with parallel detection of VSV P protein in adjacent sections, detected by immunohistochemistry; arrowheads indicate Ifit2-positive, VSV-negative cells surrounding the glomeruli; n = 2 mice. All infections were intranasal with 4×10^2^ pfu of VSV. Asterisks indicate statistical significance: ** p<0.005, *** p<0.0005; n.s.: not significant.

The protective effect of type I IFN signaling and in particular, *Ifit2*, against VSV neuropathogenesis prompted us to confirm its expression in OB and brain of wt mice, and whether it was induced in a type I IFN-dependent manner. In wt OB, *Ifit2*, *Ifit1*, and *IFN-β* mRNA was induced strongly by 2 d.p.i., and *Ifit2 and Ifit1* RNA remained abundant until day 6 d.p.i. (Figure 5A). The induction of these genes was dependent on type I IFN receptor in OB as well as in brain (Figure 5B and 5E, and data not shown). Furthermore, expression of *Ifit2* mRNA in wt OB coincided with the presence of detectable levels of the encoded Ifit2 protein ( = P54) at 2 d.p.i. and 6 d.p.i., as seen by immunohistochemistry (Figure 5C, and data not shown). Ifit2 protein staining was observed in VSV-infected cells within OB glomeruli as well as in surrounding and distant viral antigen-free cells, consistent with a remote IFN-dependent induction of Ifit2 expression (Figure 5C, *arrowheads in magnified images of right panel*). *Ifit1* and *IFN-β* mRNAs were induced as strongly in OB of *Ifit2−/−* as in wt mice, which correlated well with similar abundance of VSV RNA in wt and *Ifit2* ^−/−^ OB (Figure 5A compared to Figure 3C). In brains, at 6 d.p.i., in contrast to OB, induction of *Ifit1* and *IFN-β* mRNAs was considerably stronger in *Ifit2* ^−/−^ mice compared to wt mice (Figure 5D, 5-fold and 27-fold, respectively, both p<0.005). The enhanced gene induction in VSV-infected *Ifit2* ^−/−^ mice was not restricted to specific regions of the brain (Figure S2). Enhanced cellular gene expression also was observed for several virus-induced cytokine and chemokine genes, as measured by quantitative RT-PCR (Figure S3A). Gene expression profiling of brain tissue at day 6 d.p.i., using microarray analysis, revealed that many other genes, including ISGs, were also more strongly induced (Table S1). These results demonstrated that enhanced virus replication in the brains of *Ifit2* ^−/−^ mice led to enhanced type I IFN, other cytokines and ISG induction, which nevertheless failed to restrict VSV replication in the absence of *Ifit2*.

### Wt mice are as susceptible as *Ifit2^−/−^* mice to intracranial VSV infection

**Figure 6:**
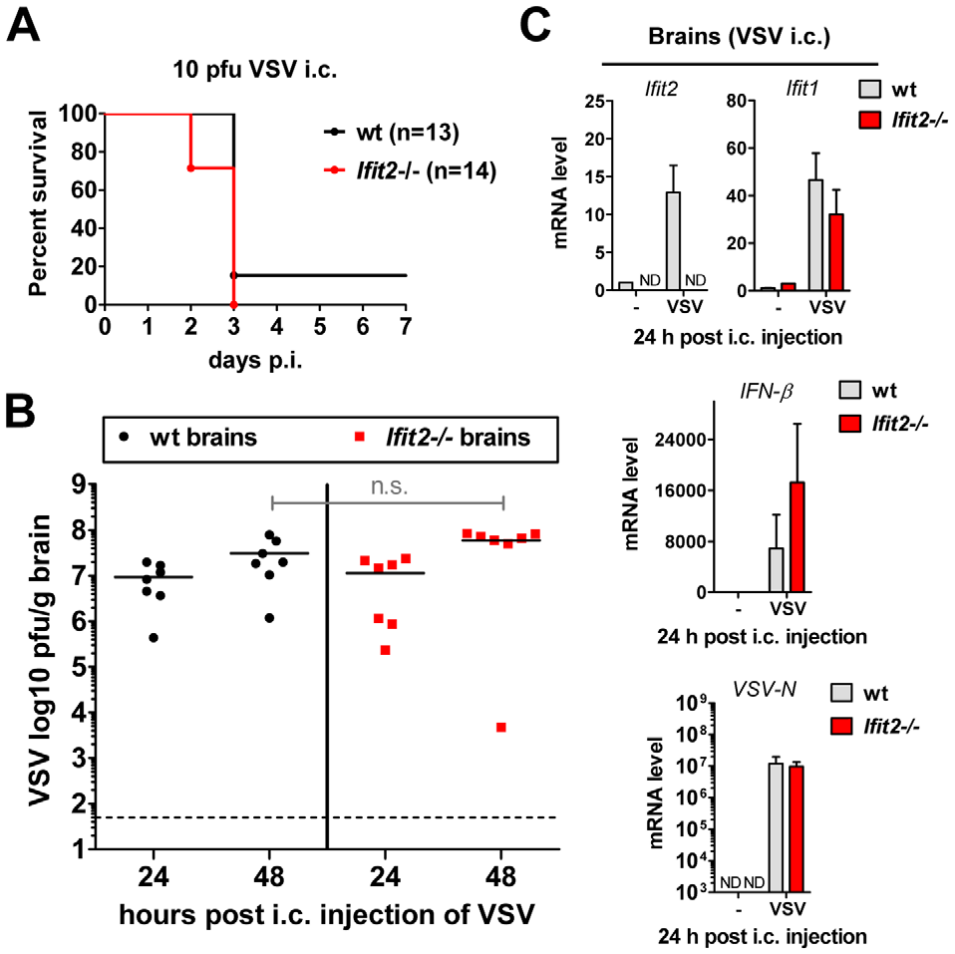
Wt mice are as susceptible as *Ifit2^−/−^* mice to intracranial VSV infection. **A**, survival of wt and *Ifit2^−/−^* mice after intracranial injection of 10 pfu of VSV; cumulative data from three independent experiments. **B**, infectious VSV titers in wt and *Ifit2* ^−/−^ brains at 24 and 48 hours after intracranial injection of 10 pfu of VSV; plotted as pfu/g with mean on log scale; dashed line depicts threshold of detection; n = 7 mice per infected group from two independent experiments; n.s.: not significant. **C**, *Ifit2*, *Ifit1*, *IFN-β* and *VSV-N* RNA levels in brains of intracranially VSV-infected or PBS-injected wt and *Ifit2* ^−/−^ mice at 24 hours p.i., plotted as mean+SD, VSV RNA levels plotted on log scale; n = 3 mice per infected group; ND, none detected.

Our results from intranasal VSV infection indicated that *Ifit2* induction in the brain was mediated by type I IFN that was, in all likelihood, produced by infected cells in the OB (Figure 5A). Virus replication and resultant IFN induction at 2 d.p.i. were similar in the OBs of wt and *Ifit2^−/−^* mice (Figs. 3C, 3D and 5A); presumably, the newly produced IFN diffused into the rest of the brain and induced local *Ifit2* expression in the wt mouse brains, prior to the arrival of the infectious virus. If this were the case, one would anticipate that direct infection of the brain, without prior action of IFN produced in infected OB, would minimize the difference between the phenotypes of wt and *Ifit2* ^−/−^ mice. To test this idea, we injected a very low dose (10 pfu) of VSV intracranially. As hypothesized, wt and *Ifit2^−/−^* mice were now equally susceptible; almost all mice died by 3 d.p.i. even at this low dose (Figure 6A) and there were equally high virus titers and viral RNA levels in the brains of mice of both genotypes (Figure 6B and 6C). Concomitant with virus replication, there was similar induction of *Ifit1* and *IFN-β* (Figure 6C) and other cytokines and chemokines (Figure S3B). These results indicate that in the absence of prior induction of *Ifit2* by IFN, brain neurons are highly susceptible to VSV infection.

### Unlike the brain, other organs of *Ifit2^−/−^* mice are not more susceptible to intranasal VSV infection

**Figure 7:**
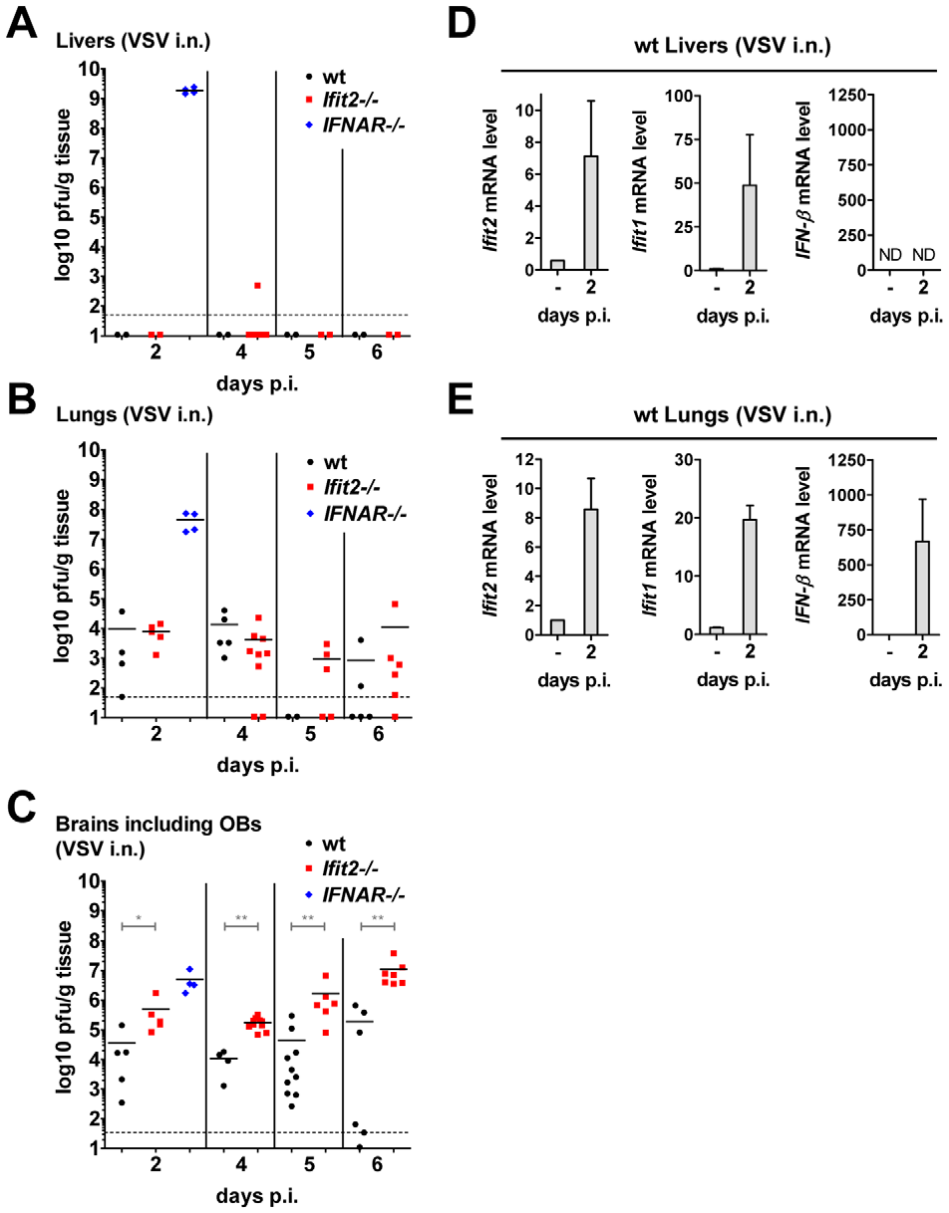
Unlike the brain, other organs of *Ifit2^−/−^* mice are not more susceptible to intranasal VSV infection. **A–C**, infectious VSV titers in organs of wt, *Ifit2* ^−/−^ or *IFNAR* ^−/−^ mice (n = 4–10 mice per group accumulated from three independent experiments) at 2, 4, 5 and 6 days after intranasal infection; livers (A), lungs (B) and brains (C, incl. OB) of the same mice were assayed and plotted as pfu/g with mean; not all available livers were titered. Dashed line depicts threshold of detection. **D/E**, *Ifit2*, *Ifit1*, *IFN-β* mRNA levels in livers (D) and lungs (E) of uninfected or VSV-infected wt mice at 2 d.p.i., plotted as mean+SD; n = 4 mice per infected group; ND, none detected. All infections were intranasal with 4×10^2^ pfu of VSV. Asterisks indicate statistical significance: * p<0.05, ** p<0.005.

*IFNAR* ^−/−^ mice succumbed within two days after VSV infection without accumulating very high VSV RNA levels in the brain (Figure 4B). These mice did not develop CNS-related signs of disease, but showed severe lethargy before death, suggesting that death was due to efficient replication of the virus in peripheral organs, due to the absence of an otherwise effective type I IFN-mediated antiviral protection of the same organs in wt mice. To test this, we assessed the kinetics of VSV accumulation in brains, livers and lungs of wt, *IFNAR* ^−/−^ and *Ifit2* ^−/−^ mice (Figure 7). At 2 d.p.i., VSV titers were very high in the liver of *IFNAR* ^−/−^ mice, reaching 10^9^ pfu/g (Figure 7A). In contrast, no or little infectious virus was detected in the liver of wt mice at 2 or 6 d.p.i., indicating efficient IFN-dependent suppression of VSV replication; intriguingly, this was also observed in *Ifit2* ^−/−^ mice, demonstrating that *Ifit2* did not mediate the anti-VSV effects of type I IFN in the liver. In lungs, which directly received a part of the virus inoculum from intranasal inhalation of VSV, the virus also replicated efficiently in *IFNAR* ^−/−^ mice, reaching 10^8^ pfu/g before death (Figure 7B). In comparison, lungs of wt and *Ifit2* ^−/−^ mice exhibited much lower levels of VSV at 2 and 4 d.p.i. (3,000 to 10,000-fold lower for wt and *Ifit2* ^−/−^ compared to *IFNAR* ^−/−^ mice, all p<0.05). By days 5 and 6 d.p.i., the virus was cleared from the lungs of a subset of wt and *Ifit2* ^−/−^ mice. In contrast, in brains from the same animals, 10 to 100-fold higher average titers (p<0.05) of VSV accumulated in *Ifit2* ^−/−^ compared to wt mice at all time points between 2 and 6 d.p.i. (Figure 7C). As expected, in wt mice, both *Ifit1* and *Ifit2* were induced not only in brains (Figure 5D), but also in livers (Figure 7D) and lungs (Figure 7E); *IFN-β* was also induced in lungs, but not livers. *Ifit1*, *Ifit2* and *IFN-β* mRNAs were also induced in the brains of EMCV-infected wt mice (Figure S3C). These findings demonstrate an unexpected brain-restricted and virus-restricted function of *Ifit2* in the context of the type I IFN-mediated antiviral response to VSV infection. They also indicate that in *Ifit2* ^−/−^ mice, other ISGs, which presumably protect the peripheral organs of VSV-infected wt mice, are either not induced in neurons or insufficient to protect them.

## Discussion

IFNs are defined by their antiviral activities. They inhibit the replication of many, if not all, viruses mostly by direct inhibition of replication in the infected cells but also by promoting the ability of immune cells to recognize and eliminate the virus-infected cells [25]. The direct effects are mediated by ISGs, which number in the hundreds, and different ISGs are thought to have more potent antiviral activities toward different families of viruses [13]. However, in most cases, it is not known which ISG inhibits the replication of a given virus; the rare exception is the Mx-mediated inhibition of influenza viruses, the underlying effect which allowed for the discovery of IFNs [26]. The task of connecting a specific IFN-induced protein to a specific antiviral action is compounded by the fact that often several IFN-induced proteins act in concert to inhibit the same virus at different stages of its life cycle. Moreover, a specific IFN-induced protein may be more relevant for inhibiting a virus in one specific cell-type than another. Recent systematic investigation of the specific antiviral effects of different ISGs has started providing significant insight into this problem [14]. Such findings are complemented by the analyses of the spectra of the antiviral effects of a specific ISG or a family of ISGs [27]. We have undertaken an investigation of the *Ifit* family of mouse ISGs. The corresponding human proteins are known to have antiviral activities against human papillomavirus (HPV) and hepatitis C virus (HCV), neither of which replicate in mouse cells. The anti-HPV activity of human IFIT1 ( = P56) has been attributed to its ability to bind HPV E1 protein and to inhibit its helicase activity, which is essential for HPV DNA replication [28], [29]. The antiviral effect on HCV, on the other hand, is manifested at the level of inhibiting viral protein synthesis as a consequence of the ability of IFIT1 to bind the translation initiation factor eIF3 and inhibit its various actions in translation initiation [30]. It has been reported recently that the IFIT1 protein can form a complex and bind to RNAs with triphosphorylated 5′ ends, presumably providing another means to inhibit specific viruses that produce such RNAs [18].

The *Ifit* genes are clustered at a single locus in both human and mouse. In the latter species, two alleles of *Ifit3* genes are flanked on two sides by one allele of *Ifit2* and one allele of *Ifit1* [15]. To identify their physiological functions, we have separately deleted the entire coding regions of *Ifit1* or *Ifit2* genes. The *Ifit1^−/−^* mice exhibited an interesting phenotype in allowing the replication of and resultant pathogenesis by a WNV mutant, which failed to replicate in wt mice [20]. Because this mutant is defective in 2′-*O* methylation of the cap structure of viral mRNAs, its rescue in the *Ifit1^−/−^* mouse indicates that this antiviral protein recognizes the 5′ ends of mRNAs, a conclusion that is consistent with the observation that, *in vitro*, it can bind to RNAs having specific structures at the 5′ ends [18]. It remains to be seen whether the proposed property of Ifit proteins to recognize 5′ ends of RNA is connected in any way to their ability to inhibit the functions of eIF3 [16], which participates in several steps of translation initiation taking place at or near the 5′ ends of mRNAs.

Replication of VSV is highly sensitive to the antiviral activity of IFNs, and VSV is widely used to determine the specific activities of IFN preparations quantitatively [21]. In spite of this strong connection, it is unclear how IFN inhibits VSV replication. An early report indicated that viral primary transcription is inhibited by IFN, but it is not known which IFN-induced protein mediates this inhibition [31]. The observed sensitivity of VSV replication *in vitro* is reflected *in vivo*. *IFNAR* ^−/−^ mice are extremely susceptible to VSV infection; they rapidly die within 2 days after infection and the virus replicates to very high titers in many organs of the infected mice. The extreme sensitivity of *IFNAR* ^−/−^ mice to VSV infection suggests that type I IFN provides the majority, if not all, of the protective innate immune defense. Eventually, protection may be facilitated by immune cell-mediated antiviral actions, but this is a slow process that does not appear to function before 6–10 days post-infection [32], [33]. Thus, it is likely that one or more ISGs directly inhibit VSV replication *in vivo*. In this context, it has been reported that mice lacking PKR, a well-studied ISG, display higher susceptibility to VSV pathogenesis [34]. However, detailed investigation of the underlying mechanism revealed that PKR did not execute IFN's antiviral action; rather, it was required for efficient induction of IFN-α/β in the infected mice [35]. *In vivo* VSV-infection induces IFN synthesis in many cell types, using either the cytoplasmic RIG-I pathway or the endosomal TLR7 pathway [4], [36]; however, it is unclear how PKR aids this process.

Our results show that *Ifit2^−/−^* mice are highly susceptible to intranasal VSV infection and the effect is gene dosage-dependent: *Ifit2^+/−^* mice had an intermediate susceptibility phenotype. Infected *Ifit2^−/−^* mice displayed symptoms of severe neuropathogenesis late after VSV infection accompanied by efficient replication of the virus in many regions of the brain. However, virus replication was restricted to neurons and did not spread to other types of cells in the brain, such as astrocytes. Our results are consistent with the hypothesis that prior, IFN-induced, *Ifit2* expression in the brain restricts VSV replication. Supporting genetic evidence for the requirement of IFN action is provided by the high susceptibility of the *IFNAR^−/−^* mice, which possess the functional *Ifit2* gene but *Ifit2* is not induced by VSV infection because these mice cannot respond to type I IFN. Additional evidence comes from a previous study using brain-specific *IFNAR^−/−^* mice, which displayed a pattern of susceptibility to intranasal VSV infection similar to that of our *Ifit2^−/−^* mice [1]. In our experimental system, the source of the IFN production was most likely the OBs; abundant IFN was induced there early (2 d.p.i.) after infection (Figure 5A) causing the induction of *Ifit2* in wt mice (Figure 5C). *Ifit2* was also induced at this time in the rest of the brain, without any induction of *IFN* mRNA (Figure 5D) suggesting that the source of IFN was the OB. In accord with the well-established concept of IFN action, pre-induction of *Ifit2* in neurons, before the onset of infection, was essential for the antiviral effect. In comparison, induction of *IFN* and *Ifit2* that was concomitant with VSV infection failed to have an appreciable antiviral effect, as manifested by robust virus replication at directly infected sites, such as the OBs of wt mice infected intranasally (Figure 3D) or the brain of wt mice infected intracranially (Figure 6B). High mortality of the infected mice correlated with high virus titers in the brains of intranasally infected *Ifit2* ^−/−^ mice or intracranially infected wt and *Ifit2^−/−^* mice. In the intranasally infected *Ifit2^−/−^* mice, death was not preceded by widespread apoptosis in the brain (Figure S4). However, as expected with high viral loads, IFN and other cytokines and chemokines were strongly induced (Figures 5D, S2 and S3A); consequently, many ISGs, except *Ifit2*, were also induced (Table S1).

Pre-induced *Ifit2* prevents efficient VSV replication in the brain, most probably by blocking one or more essential step of the viral life cycle including viral entry, uncoating, primary transcription, viral protein synthesis, RNA replication, virion assembly or egress. It also might block trans-synaptic spread of the virus, although unlike another rhabdovirus, rabies virus, VSV is not known to depend on transit from neuron to neuron. In this context, it is important to note the observations made by Iannacone et al. [37] using a footpad VSV infection model. They concluded that type I IFN, produced by infected macrophages and plasmacytoid dendritic cells in infected mice, blocked infection of peripheral neurons resulting in lowered infection of the CNS and prevention of neuropathogenesis. It is worth noting that in our studies, the absence of *Ifit2* did not affect IFN induction by VSV (Figures 5A and 6C). Further investigation of the biochemical mechanism behind the observed *in vivo* effect of *Ifit2^−/−^* is hampered by the absence of a suitable cell culture model of the phenomenon. For example, *Ifit2* was not required for mediating the anti-VSV effect of IFN in mouse embryonic fibroblasts (Figure S5), in primary fetal neurons or in *Ifit2*-ablated neuroblastoma cells (data not shown), results that are not surprising given the strong tissue-specificity of *Ifit2* action observed *in vivo* (Figure 7). Specific RNA-binding properties of Ifit proteins have been recently reported [18]. Following this lead, we examined the RNA-binding properties of recombinant murine Ifit1 and Ifit2 using VSV leader RNA as the probe in an electrophoretic mobility shift assay: Ifit1 bound RNA with a 5′-ppp end but not with a 5′-OH end; however, Ifit2 bound neither (Figure S6). To obtain meaningful leads, future investigation of this kind may require using brain extracts from infected mice to detect protein-viral RNA complexes that may contain Ifit2 along with adult neuron-specific proteins.

Our results revealed several layers of specificity of IFN action, some of which were not anticipated. First, compared to *Ifit2^−/−^* mice, *Ifit1^−/−^* mice were much less susceptible to intranasal VSV infection; this was true for both low and high doses of virus. This finding was surprising in view of a recent report on VSV susceptibility of *Ifit1^−/−^* mice [18] and the observation that Ifit1, but not Ifit2, could bind VSV leader RNA *in vitro* (Figure S6). The above results demonstrate that different Ifit proteins have non-redundant functions *in vivo*. The second layer of specificity was directed toward the nature of the infecting virus. Although both VSV and EMCV caused neuroinvasive disease, induced *IFN-β*, *Ifit1* and *Ifit2* in the brain and type I IFN action was required for protection against both viruses, *Ifit2* was critical only for protection against VSV; the absence of either *Ifit1* or *Ifit2* did not exacerbate susceptibility to EMCV. The third layer of specificity was revealed by the organ-specific action of *Ifit2*. In the complete absence of type I IFN action in the *IFNAR^−/−^* mice, intranasally infected VSV replicated vigorously not only in brains, but also in livers and lungs (Figure 7A–C). In contrast, in *Ifit2^−/−^* mice, efficient VSV replication was restricted to the brain suggesting that *Ifit2* does not act as an anti-VSV ISG in the liver or the lung because its absence did not impact virus titers, even though *Ifit2* was induced in these organs of infected wt mice (Figure 7D and 7E). The efficient VSV replication in livers and lungs of *IFNAR^−/−^* mice, but not wt and *Ifit2^−/−^* mice, indicates that other ISGs must have anti-VSV effects in those organs. Further investigation is needed to determine the basis of neuronal specificity of *Ifit2* action and the identities of other ISGs that inhibit VSV replication in other organs.

## Materials and Methods

### Ethics statement

All animal experiments were performed in strict accordance with all provisions of the Animal Welfare Act, the Guide for the Care and Use of Laboratory Animals, and the PHS Policy on Humane Care and Use of Laboratory Animals. The protocol was approved by the Cleveland Clinic Institutional Animal Care and Use Committee (IACUC), PHS Assurance number A3047-01. All experimental manipulations or intranasal instillations of mice were performed under anesthesia induced by pentobarbital sodium or isofluorane, respectively, and all efforts were made to minimize suffering.

### Mice

All mice used were of C57BL/6 background and of both sexes; *Ifit2* ^−/−^ mice were custom-generated by Taconic Farms, Inc. by flanking exons 2 and 3 of *Ifit2*, encompassing the complete protein-encoding region, with *frt* sites in C57BL/6 embryonic stem (ES) cells, and deleting the flanked region by transfection of Flp recombinase. ES cell clones were injected into BL/6 blastocysts, and heterozygous offspring mice were crossed to homozygosity. *Ifit1* ^−/−^ mice were generated from C57BL/6 ES cells lacking the whole coding region of *Ifit1* (20); ES cells were obtained from the NIH Knockout mouse project (KOMP, allele *Ifit1* ^tm1(KOMP)Vlcg^). The same ES cell line was independently used to generate mice in another study [18]. *IFNAR^−/−^* mice (lacking *Ifnar1*) were a gift of Murali-Krishna Kaja (Emory University, Atlanta, GA). Congenic wild-type mice were obtained from Taconic Farms.

### Viruses and infections

Vesicular stomatitis virus (VSV) Indiana was a gift from Amiya K. Banerjee, Lerner Research Institute, Cleveland, Ohio. For intranasal infections, between 4×10^2^ and 4×10^6^ pfu of VSV in 35 µl of endotoxin-free PBS were inhaled by isofluorane-anesthetized 8–12 week-old mice, with PBS-only as control. For intracranial infections, 10 pfu of VSV in 30 µl of endotoxin-free PBS were injected into the brains of 6–7 week-old mice, with PBS-only as control. Thereafter, mice were monitored daily (twice daily after i.c. injection) for weight loss and symptoms of disease. Encephalomyocarditis virus (EMCV) K strain was a gift from Robert H. Silverman, Lerner Research Institute, Cleveland, Ohio. For intraperitoneal infections, between 25 and 5×10^2^ pfu of EMCV in 500 µl of PBS were injected into the peritoneal cavity of mice. Mice were monitored daily for weight loss and symptoms of disease.

### Immunohistochemistry and TUNEL assay

Mice were anesthetized with pentobarbital (150 mg/kg) and blood was removed from organs by cardiac perfusion with 10 ml of PBS, followed by perfusion with 10 ml of 4% paraformaldehyde/PBS for fixation. Brains were placed in 4% paraformaldehyde overnight for complete fixation, submerged in 30% sucrose/PBS overnight for cryoprotection, and frozen in O.C.T. compound (Sakura Finetek USA, Torrance, CA, USA). 10 µm sagittal sections were cut at −20°C in a Leica CM1900 cryostat, mounted on coated slides (Superfrost Plus, Fisherbrand, Fisher Scientific); membranes were permeabilized by 0.2% Triton X-100/PBS treatment for 15 min. For immunohistochemistry, the Envision+ DAB kit (Dako, Carpinteria, CA) was used with anti-mouse Ifit2/P54 [38] or anti-VSV-P protein (a gift from Amiya K. Banerjee, Lerner Research Institute, Cleveland, Ohio) as primary antibodies. For immunohistofluorescence, anti-VSV-P or anti-NeuN (Chemicon Intl./Millipore, Billerica, MA) or anti-GFAP (Sigma-Aldrich, St. Louis, MO) were used; labeled brain sections were stained with AlexaFluor-594 secondary antibody (Invitrogen/Molecular Probes, Carlsbad, CA). For detection of apoptotic cells in brain sections, the DeadEnd fluorometric TUNEL system (Promega) was used according to manufacturer's instructions. All objects were then mounted with VectaShield (with DAPI, Vector Labs, Burlingame, CA), and examined with a Leica DRM fluorescence microscope.

### Quantitative RT-PCR and microarray analysis

Mice were anesthetized with pentobarbital (150 mg/kg) and blood was removed from organs after cardiac perfusion with 10 ml of PBS. Brains were separated into olfactory bulbs and the remainder of the brain, snap-frozen in liquid nitrogen (as well as livers and lungs) and RNA was extracted using TRIzol reagent (Invitrogen). DNase I treatment (DNA*free*, Applied Biosystems/Ambion) and reverse transcription with random hexamers (ImProm-II, Promega) were performed according to manufacturer's instructions. 0.5 ng of RNA was used in 384 well-format realtime PCRs in a Roche LightCycler 480 II using Applied Biosystem's SYBR Green PCR core reagents. PCR primers for murine *ISG49/Ifit3*, *ISG54/Ifit2*, *ISG56/Ifit1* and 18S rRNA have been published previously [17]; primers targeting murine *Ifnb1* [5′-CTTCTCCGTCATCTCCATAGGG-3′ [39], with the alternative reverse primer: 5′-CACAGCCCTCTCCATCAACT-3′], *VSV N* RNA [40] or *EMCV 3D polymerase* genomic region [41] were described previously. Primers for *Ccl2*, *Il1b*, *Il6*, *Tnf*, *Il12b* and *Nos2* have been described previously [42], [43]. Average expression levels, relative to 18S rRNA and normalized by use of calibrator samples, were graphed with Prism 5.02 software. For analysis of different regions of the brain, brains without OB of perfused mice were separated into cortex, cerebellum, brain stem and remaining “midbrain”, and tissue was submerged into RNA*later* stabilizing reagent (Qiagen) overnight and frozen. RNA was then extracted via TRIzol and further processed and assayed by realtime RT-PCR as described above. For microarray analysis, TRIzol-extracted and DNase I-treated RNA was additionally purified using spin columns (RNeasy Mini kit, Qiagen) before subjection to mRNA expression microarray analysis via Illumina Mouse Ref-8 V2 beadchip and GenomeStudio software V2010.2 (Illumina, Inc.); RNA hybridization to chips was performed by the Lerner Research Institute Genomics Core at the Cleveland Clinic. Microarray raw data were deposited in the NCBI Gene Expression Omnibus (GEO), accession number GSE33678.

### Virus quantification

For quantification of infectious VSV in organs, mice were anesthetized with pentobarbital (150 mg/kg) and blood was removed from organs by cardiac perfusion with 10 ml of PBS. Organs were snap-frozen in liquid nitrogen, weighed, pestle/tube-homogenized (Kimble/Kontes) in 1 ml of PBS per brain or peripheral organ or 0.1 ml per pair of olfactory bulbs, and virus was titered in 10-fold serial dilutions on Vero cells by plaque assay. Results are expressed as plaque-forming units (pfu) per gram of tissue. For quantification of infectious VSV yields in MEF, cells (−/+IFN-β pretreatment as indicated) were infected with VSV inoculum for 1 h, and after another 12 h, cells were freeze/thawed, and cleared supernatants of lysates were assayed for VSV by plaque assay on Vero cells.

### Immunoblot

Primary murine embryonic fibroblasts (MEFs) were stimulated with 1000 U/ml murine IFN-β (PBL, Inc., Piscataway, NJ) for 16 h and lysed in lysis buffer [50 mM Tris pH 7.6, 150 mM NaCl, 0.5% Triton X-100, 1 mM sodium orthovanadate, 10 mM sodium fluoride, 5 mM sodium pyrophosphate, 10 mM β-glycerophosphate and 1× complete EDTA-free protease inhibitor (Roche, Indianapolis, IN)]. 10 µg of whole cell extract were separated via 10% SDS-PAGE, transferred to PVDF membranes, blocked with 5% dry milk in Tris-buffered saline/0.05% Tween-20 overnight and labeled with anti-Ifit3/P49, anti-Ifit2/P54 or anti-Ifit1/P56 polyclonal rabbit sera [17], [38].

### Electrophoretic mobility shift assay

Single-stranded VSV leader RNA (nucleotides 1–18) was T7 polymerase-transcribed in presence of [α-^32^P]-CTP, yielding radiolabeled 5′-triphosphorylated (ppp-) RNA, followed by alkaline phosphatase treatment for generation of 5′-hydroxyl (HO-) RNA. ppp-RNA or HO-RNA were added to bacterially expressed and purified 6xHis-tagged Ifit1 or Ifit2 protein in reaction buffer (50 mM Tris pH 8.0, 100 mM NaCl, 1 mM EDTA, 2 mM DTT, 0.05% Triton X-100, 10% glycerol) and incubated for 30 min on ice. Reaction products were separated by 6% native polyacrylamide gel electrophoresis followed by exposure to film.

### Statistical analysis

Statistical significance of mouse survival differences was calculated by Mantel-Cox log rank test. To assess significance of differences in gene expressions or virus titers, the two-tailed Mann-Whitney test was used. All calculations were performed using GraphPad Prism 5.02 software.

### Gene accession numbers

Previously published transcript sequences in the NCBI Entrez Nucleotide database: *Ifit2*, NM_008332; *Ifit1*, NM_008331; *Ifit3*, NM_010501; *Ifnb1*, NM_010510; *Ifnar1*, NM_010508.

## Supporting Information

Figure S1 **Survival of wt and** ***Ifit2^−/−^*** **mice after infection with low EMCV dose (25 pfu).** Statistical significance of survival differences is indicated by p-value; n.s., not significant.
(PDF)

Figure S2 **Enhanced** ***ISG*** **and** ***IFN-β*** **induction in intranasally VSV-infected** ***Ifit2*** **^−/−^ brain regions.** *IFN-β-*, and *Ifit3/2/1* mRNA levels in different regions of brains of uninfected or VSV-infected wt and *Ifit2* ^−/−^ mice at 6 d.p.i., plotted as mean+SD. n = 4 mice per infected group; ND, not done. Infections were intranasal with 4×10^2^ pfu of VSV.
(PDF)

Figure S3 **Gene induction in brains after VSV or EMCV infections.** **A**, mRNA levels of select genes in brains (without OBs) of uninfected or intranasally VSV-infected wt and *Ifit2* ^−/−^ mice at 6 d.p.i., plotted as mean+SD; n = 3 mice per infected group; infection was intranasal with 4×10^2^ pfu of VSV. **B**, mRNA levels of select genes in brains (without OBs) of uninfected or intracranially VSV-infected wt and *Ifit2* ^−/−^ mice at 24 h post injection, plotted as mean+SD; n = 4 mice per infected group; infection was intracranial injection with 10 pfu of VSV. **C**, *Ifit2*, *Ifit1*, *IFN-β* and EMCV RNA levels in brains 4 days after EMCV infection (5×10^2^ pfu, n = 3 mice per infected group).
(PDF)

Figure S4 **Region-selective induction of apoptosis in brains of intranasally VSV-infected** ***Ifit2^−/−^*** **mice.** *Ifit2^−/−^* mice were i.n. infected with 4×10^2^ pfu of VSV; at 6 d.p.i., adjacent sections of fixed brains were labeled to detect apoptotic cells (TUNEL) or VSV P protein (immunohistofluorescence), n = 2 mice; only few regions such as striatum show positive TUNEL; infected wt brains and uninfected control brains of either genotype did not show appreciable signals, hence data not shown).
(PDF)

Figure S5 **VSV yields from infected wt and** ***Ifit2^−/^*** ^**−**^ **MEF.** Immortalized MEF were treated for 16 h with 10 U/ml IFN-β and infected with VSV at moi 10. 12 hours after infection, virus yields were determined by plaque assay. Results are plotted as mean+SD on log scale, representing one of two independent experiments.
(PDF)

Figure S6 **Murine Ifit2 protein does not bind ppp-RNA.** Single-stranded radiolabeled VSV leader RNAs (nt 1–18) with either 5′-triphosphorylated or free 5′-hydroxyl-ends (ppp-RNA or HO-RNA) were *in vitro* incubated with purified murine Ifit1 ( = P56) or Ifit2 ( = P54) proteins; formation of protein/RNA complex was detected by electrophoretic mobility shift assay.
(PDF)

Table S1 **Enhanced gene expression in brains incl. OBs of intranasally VSV-infected** ***Ifit2*** **^−/−^ versus wt mice at 6 d.p.i.** Wt or *Ifit2* ^−/−^ mice were intranasally VSV-infected with 4×10^2^ pfu, and at 2 or 6 d.p.i., brain (incl. OB) RNA expression profiles were obtained by microarray. Genes are ranked by their “fold expression level in *Ifit2* ^−/−^ over wt at 6 d.p.i.”. Only genes with at least 3-fold higher expression level in *Ifit2* ^−/−^ are included. Note: The *Ifit1/ISG56* probe of the Illumina mouse Ref-8 chip is defective and therefore the gene is not included in this list.
(PDF)
