# Supplementary material for: Interferon-Induced Ifit2/ISG54 Protects Mice from Lethal VSV Neuropathogenesis
Source: PLoS Pathog. 2012 May 17;8(5):e1002712. doi: 10.1371/journal.ppat.1002712 (PMC3355090; doi:10.1371/journal.ppat.1002712)
Supplement: Figure S1 — Survival of wt and Ifit2−/− mice after infection with low EMCV dose (25 pfu). Statistical significance of survival differences is indicated by p-value; n.s., not significant. (PDF) [file ppat.1002712.s001.pdf]

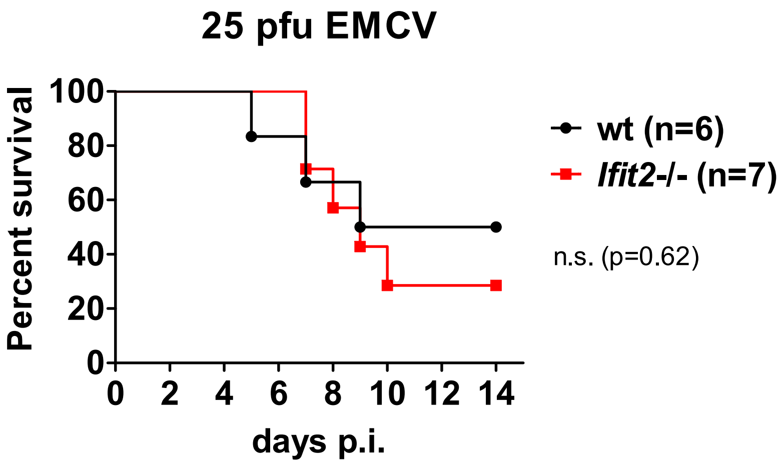

Figure S1. Survival of wt and *Ifit2*$^{-/-}$ mice after infection with low EMCV dose (25 pfu). Statistical significance of survival differences is indicated by p-value; n.s., not significant.
