# Supplementary material for: Interferon-Induced Ifit2/ISG54 Protects Mice from Lethal VSV Neuropathogenesis
Source: PLoS Pathog. 2012 May 17;8(5):e1002712. doi: 10.1371/journal.ppat.1002712 (PMC3355090; doi:10.1371/journal.ppat.1002712)
Supplement: Figure S2 — Enhanced ISG and IFN-β induction in intranasally VSV-infected Ifit2 −/− brain regions. IFN-β-, and Ifit3/2/1 mRNA levels in different regions of brains of uninfected or VSV-infected wt and Ifit2 −/− mice at 6 d.p.i., plotted as mean+SD. n = 4 mice per infected group; ND, not done. Infections were intranasal with 4×10^2^ pfu of VSV. (PDF) [file ppat.1002712.s002.pdf]

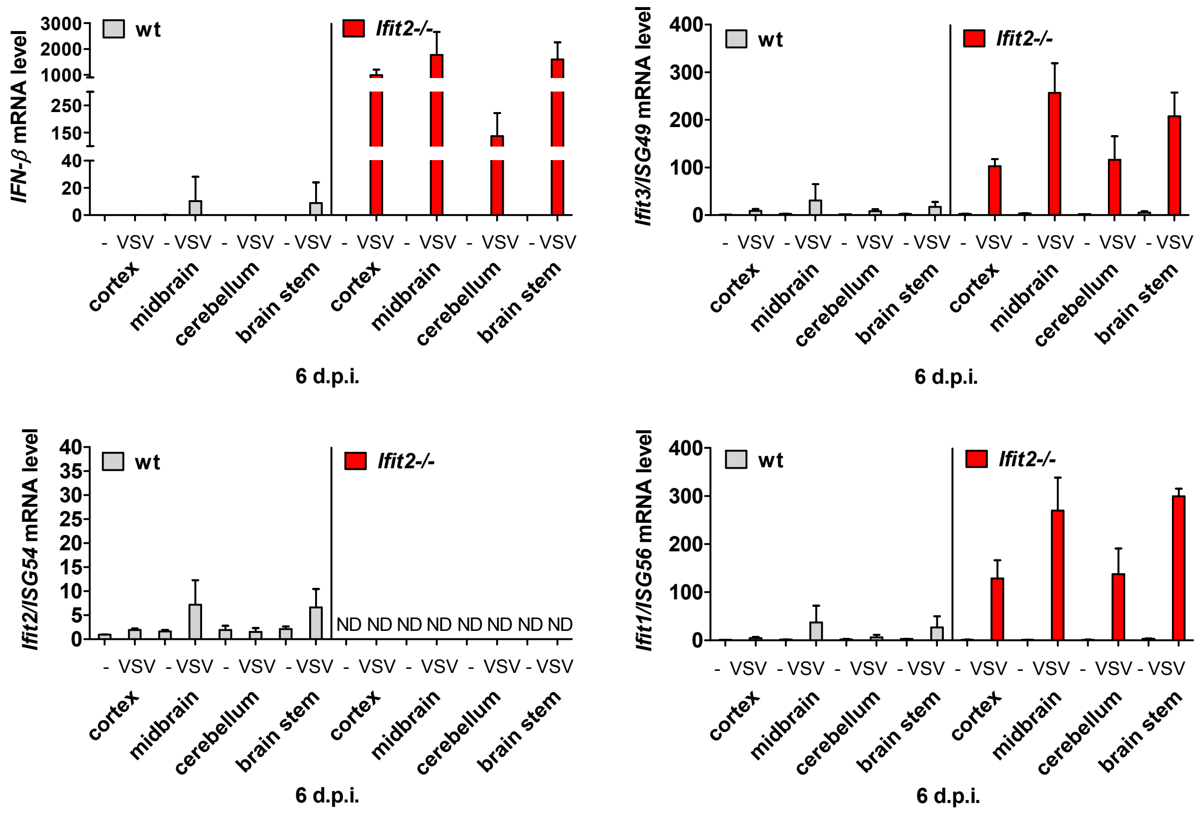

Figure S2. Enhanced *ISG* and *IFN-β* induction in intranasally VSV-infected *Ifit2*$^{-/-}$ brain regions. *IFN-β*-, and *Ifit3/2/1* mRNA levels in different regions of brains of uninfected or VSV-infected wt and *Ifit2*$^{-/-}$ mice at 6 d.p.i., plotted as mean+SD. n=4 mice per infected group; ND, not done. Infections were intranasal with $4x10^2$ pfu of VSV.
