# Supplementary material for: Interferon-Induced Ifit2/ISG54 Protects Mice from Lethal VSV Neuropathogenesis
Source: PLoS Pathog. 2012 May 17;8(5):e1002712. doi: 10.1371/journal.ppat.1002712 (PMC3355090; doi:10.1371/journal.ppat.1002712)
Supplement: Figure S3 — Gene induction in brains after VSV or EMCV infections. A, mRNA levels of select genes in brains (without OBs) of uninfected or intranasally VSV-infected wt and Ifit2 −/− mice at 6 d.p.i., plotted as mean+SD; n = 3 mice per infected group; infection was intranasal with 4×10^2^ pfu of VSV. B, mRNA levels of select genes in brains (without OBs) of uninfected or intracranially VSV-infected wt and Ifit2 −/− mice at 24 h post injection, plotted as mean+SD; n = 4 mice per infected group; infection was intracranial injection with 10 pfu of VSV. C, Ifit2, Ifit1, IFN-β and EMCV RNA levels in brains 4 days after EMCV infection (5×10^2^ pfu, n = 3 mice per infected group). (PDF) [file ppat.1002712.s003.pdf]

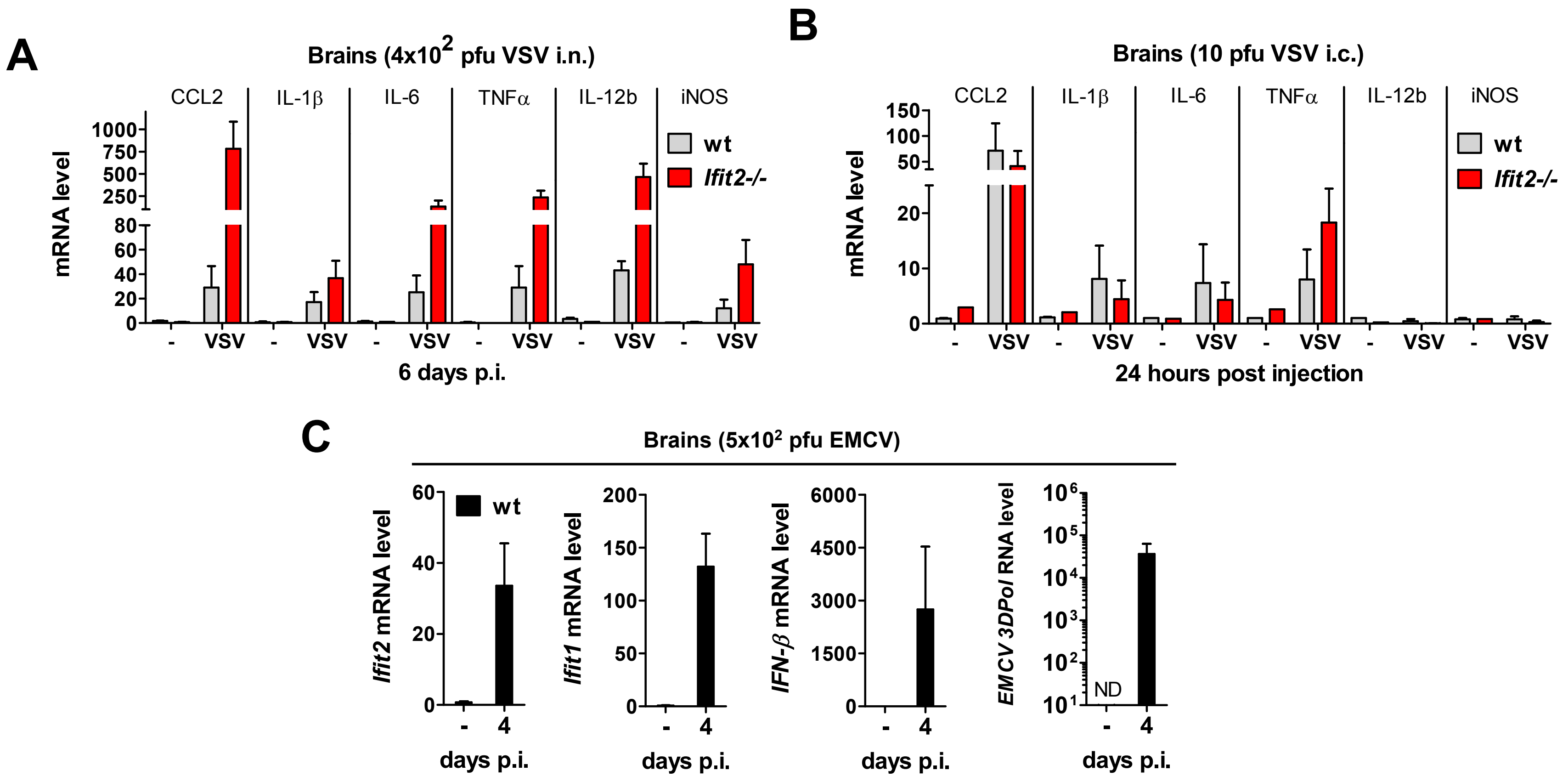

Figure S3. Gene induction in brains after VSV or EMCV infections. ***A***, mRNA levels of select genes in brains (without OBs) of uninfected or intranasally VSV-infected wt and *Ifit2*$^{-/-}$ mice at 6 d.p.i., plotted as mean+SD; n=3 mice per infected group; infection was intranasal with 4x10^2 pfu of VSV ***B***, mRNA levels of select genes in brains (without OBs) of uninfected or intracranially VSV-infected wt and *Ifit2*-/- mice at 24 h post injection, plotted as mean+SD; n=4 mice per infected group; infection was intracranial injection with 10 pfu of VSV. ***C**, Ifit2, Ifit1, IFN-β* and EMCV RNA levels in brains 4 days after EMCV infection (5x10^2 pfu, n=3 mice per infected group).
