# Supplementary material for: Interferon-Induced Ifit2/ISG54 Protects Mice from Lethal VSV Neuropathogenesis
Source: PLoS Pathog. 2012 May 17;8(5):e1002712. doi: 10.1371/journal.ppat.1002712 (PMC3355090; doi:10.1371/journal.ppat.1002712)
Supplement: Figure S4 — Region-selective induction of apoptosis in brains of intranasally VSV-infected Ifit2−/− mice. Ifit2−/− mice were i.n. infected with 4×10^2^ pfu of VSV; at 6 d.p.i., adjacent sections of fixed brains were labeled to detect apoptotic cells (TUNEL) or VSV P protein (immunohistofluorescence), n = 2 mice; only few regions such as striatum show positive TUNEL; infected wt brains and uninfected control brains of either genotype did not show appreciable signals, hence data not shown). (PDF) [file ppat.1002712.s004.pdf]

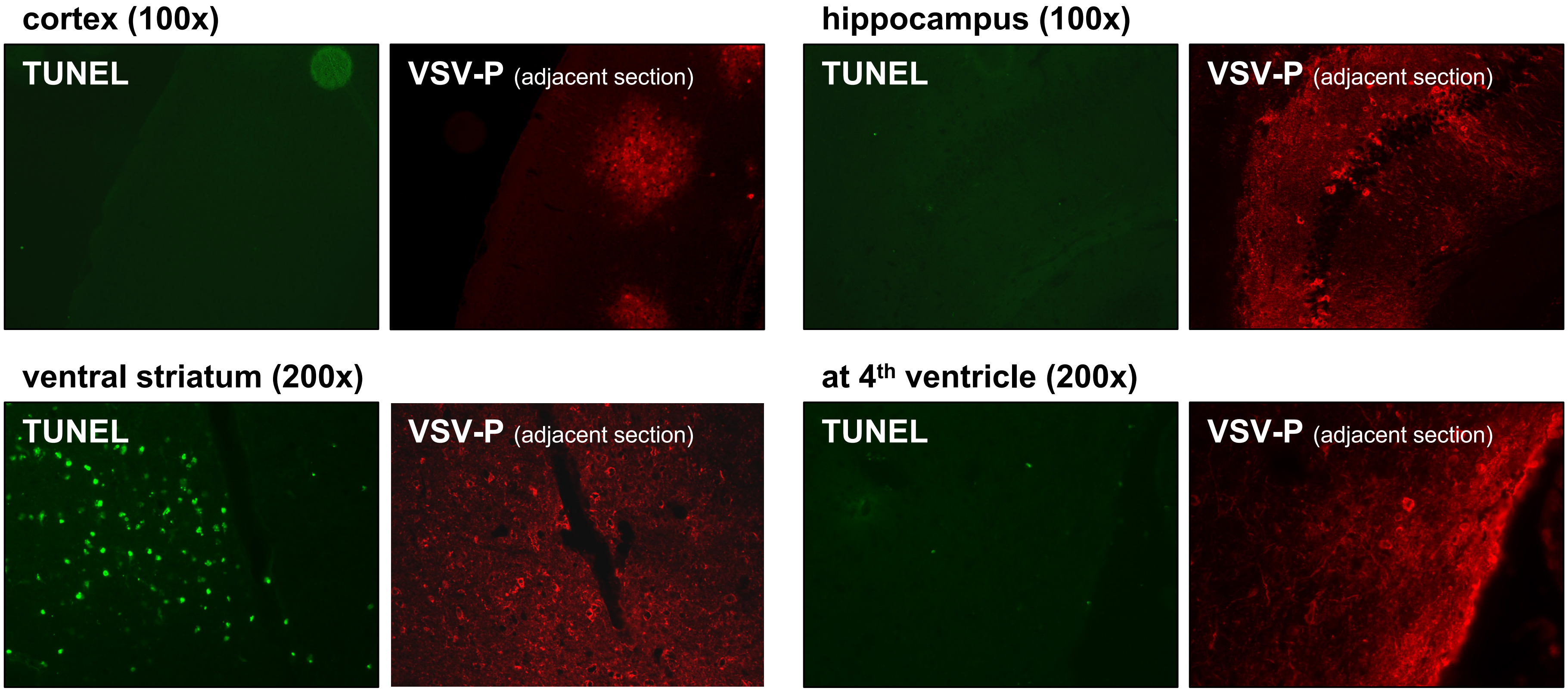

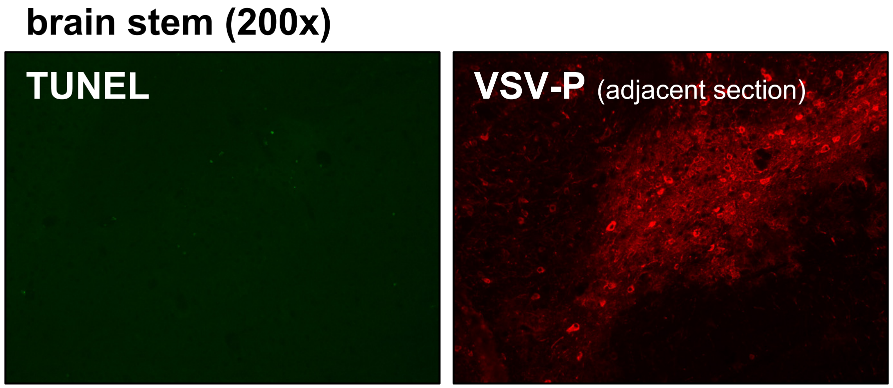

Figure S4. Region-selective induction of apoptosis in brains of intranasally VSV-infected *Ifit2*$^{-/-}$ mice. *Ifit2*$^{-/-}$ mice were i.n. infected with 4x10^2 pfu of VSV; at 6 d.p.i., adjacent sections of fixed brains were labeled to detect apoptotic cells (TUNEL) or VSV P protein (immunohisto-fluorescence), n=2 mice; only few regions such as striatum show positive TUNEL; infected wt brains and uninfected control brains of either genotype did not show appreciable signals, hence data not shown).
