# Supplementary material for: Interferon-Induced Ifit2/ISG54 Protects Mice from Lethal VSV Neuropathogenesis
Source: PLoS Pathog. 2012 May 17;8(5):e1002712. doi: 10.1371/journal.ppat.1002712 (PMC3355090; doi:10.1371/journal.ppat.1002712)
Supplement: Figure S5 — VSV yields from infected wt and Ifit2−/ − MEF. Immortalized MEF were treated for 16 h with 10 U/ml IFN-β and infected with VSV at moi 10. 12 hours after infection, virus yields were determined by plaque assay. Results are plotted as mean+SD on log scale, representing one of two independent experiments. (PDF) [file ppat.1002712.s005.pdf]

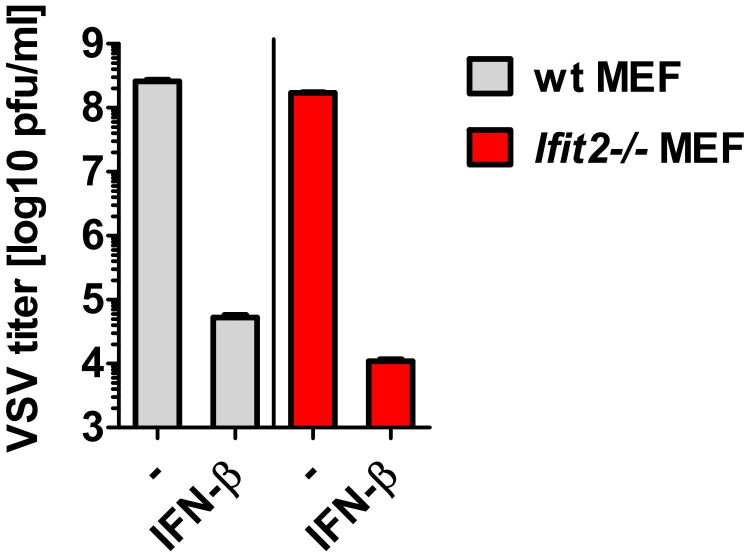

Figure S5. VSV yields from infected wt and *Ifit2*$^{-/-}$ MEF. Immortalized MEF were treated for 16 h with 10 U/ml IFN-β and infected with VSV at moi 10. 12 hours after infection, virus yields were determined by plaque assay. Results are plotted as mean+SD on log scale, representing one of two independent experiments.
