# Supplementary material for: Interferon-Induced Ifit2/ISG54 Protects Mice from Lethal VSV Neuropathogenesis
Source: PLoS Pathog. 2012 May 17;8(5):e1002712. doi: 10.1371/journal.ppat.1002712 (PMC3355090; doi:10.1371/journal.ppat.1002712)
Supplement: Figure S6 — Murine Ifit2 protein does not bind ppp-RNA. Single-stranded radiolabeled VSV leader RNAs (nt 1–18) with either 5′-triphosphorylated or free 5′-hydroxyl-ends (ppp-RNA or HO-RNA) were in vitro incubated with purified murine Ifit1 ( = P56) or Ifit2 ( = P54) proteins; formation of protein/RNA complex was detected by electrophoretic mobility shift assay. (PDF) [file ppat.1002712.s006.pdf]

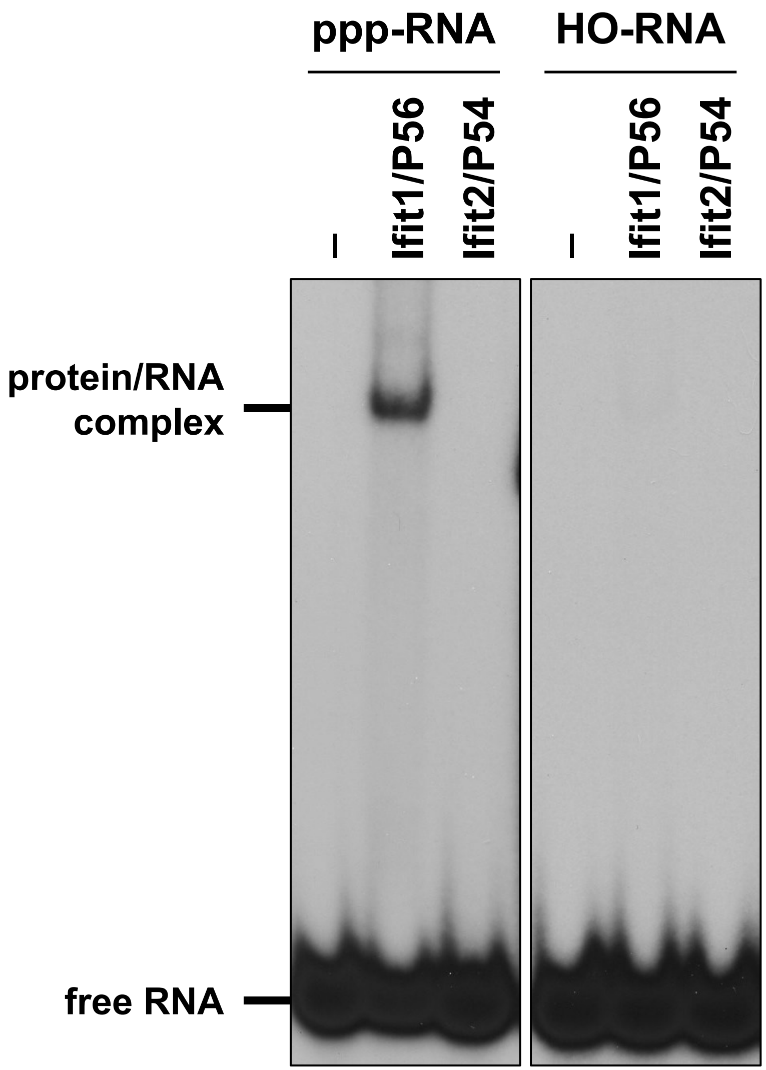

Figure S6. Murine Ifit2 protein does not bind ppp-RNA. Single-stranded radiolabeled VSV leader RNAs (nt 1-18) with either 5'-triphosphorylated or free 5'-hydroxyl-ends (ppp-RNA or HO-RNA) were *in vitro* incubated with purified murine Ifit1 (= P56) or Ifit2 (= P54) proteins; formation of protein/RNA complex was detected by electrophoretic mobility shift assay.
