# Supplementary material for: Interferon-Induced Ifit2/ISG54 Protects Mice from Lethal VSV Neuropathogenesis
Source: PLoS Pathog. 2012 May 17;8(5):e1002712. doi: 10.1371/journal.ppat.1002712 (PMC3355090; doi:10.1371/journal.ppat.1002712)
Supplement: Table S1 — Enhanced gene expression in brains incl. OBs of intranasally VSV-infected Ifit2 −/− versus wt mice at 6 d.p.i. Wt or Ifit2 −/− mice were intranasally VSV-infected with 4×10^2^ pfu, and at 2 or 6 d.p.i., brain (incl. OB) RNA expression profiles were obtained by microarray. Genes are ranked by their “fold expression level in Ifit2 −/− over wt at 6 d.p.i.”. Only genes with at least 3-fold higher expression level in Ifit2 −/− are included. Note: The Ifit1/ISG56 probe of the Illumina mouse Ref-8 chip is defective and therefore the gene is not included in this list. (PDF) [file ppat.1002712.s007.pdf]

Table S1: Enhanced gene expression in brains incl. OBs of intranasally VSV-infected *Ifit2*$^{-/-}$ versus wt mice at 6 d.p.i.

Gene symbol	Fold expression in *Ifit2*$^{-/-}$ over wt (6 d.p.i.)	Fold expression in *Ifit2*$^{-/-}$ over wt (2 d.p.i.)	Definition
Ifnb1	**77.2**	5.2	interferon beta 1, fibroblast (Ifnb1).
Isg20	**14.2**	1.7	interferon-stimulated protein (Isg20).
Ddx3y	**12.4**	1.3	DEAD (Asp-Glu-Ala-Asp) box polypeptide 3, Y-linked (Ddx3y).
Saa3	**8.2**	0.7	serum amyloid A 3 (Saa3).
Ccl2	**7.4**	4.9	chemokine (C-C motif) ligand 2 (Ccl2).
Lcn2	**6.7**	1.6	lipocalin 2 (Lcn2).
Slpi	**6.6**	1.2	secretory leukocyte peptidase inhibitor (Slpi).
Arg1	**6.0**	0.2	arginase 1, liver (Arg1).
Bst2	**5.9**	0.9	bone marrow stromal cell antigen 2 (Bst2).
Cfb	**5.8**	19.7	complement factor B (Cfb).
Hmox1	**5.5**	1.0	heme oxygenase (decycling) 1 (Hmox1).
Cxcl1	**4.8**	1.1	chemokine (C-X-C motif) ligand 1 (Cxcl1).
Gpnmb	**4.7**	1.0	glycoprotein (transmembrane) nmb (Gpnmb).
Mt2	**4.5**	0.8	metallothionein 2 (Mt2).
Hspb1	**4.3**	3.5	heat shock protein 1 (Hspb1).
Chi3l3	**4.2**	-0.4	chitinase 3-like 3 (Chi3l3).
Cdkn1a	**4.1**	1.4	cyclin-dependent kinase inhibitor 1A (P21) (Cdkn1a).
Mx1	**4.1**	0.9	myxovirus (influenza virus) resistance 1 (Mx1).
Ecm1	**4.1**	1.4	extracellular matrix protein 1 (Ecm1).
Rhoj	**4.0**	1.3	ras homolog gene family, member J (Rhoj).
Tyki	**4.0**	1.5	thymidylate kinase LPS-inducible member
Ptpre	**4.0**	4.1	protein tyrosine phosphatase, receptor type, E (Ptpre).
Ifit3/ISG49	**3.9**	1.2	interferon-induced protein with tetratricopeptide repeats 3 (Ifit3).
Srgn	**3.8**	3.3	serglycin (Srgn).
Gbp6	**3.6**	0.7	guanylate binding protein 6 (Gbp6).
Cdkn1a	**3.6**	0.7	cyclin-dependent kinase inhibitor 1A (P21) (Cdkn1a).
Sct	**3.6**	0.7	secretin (Sct).
Csf1	**3.4**	1.0	colony stimulating factor 1 (macrophage) (Csf1).
Lgals9	**3.4**	1.5	lectin, galactose binding, soluble 9 (Lgals9).
H2-Q8	**3.4**	1.5	histocompatibility 2, Q region locus 8 (H2-Q8).
Gadd45b	**3.4**	1.4	growth arrest and DNA-damage-inducible 45 beta (Gadd45b).
H2-T23	**3.4**	1.0	histocompatibility 2, T region locus 23 (H2-T23).
Rsad2	**3.3**	0.6	radical S-adenosyl methionine domain containing 2 (Rsad2).
Stat2	**3.3**	0.9	signal transducer and activator of transcription 2 (Stat2).
Map1lc3b	**3.3**	3.3	microtubule-associated protein 1 light chain 3 beta (Map1lc3b).
Cyp1b1	**3.2**	1.3	cytochrome P450, family 1, subfamily b, polypeptide 1 (Cyp1b1).
Oasl1	**3.2**	1.0	2'-5' oligoadenylate synthetase-like 1 (Oasl1).
Ctss	**3.2**	1.2	cathepsin S (Ctss).
Trim56	**3.2**	1.2	tripartite motif-containing 56 (Trim56).
Ube2l6	**3.2**	0.7	ubiquitin-conjugating enzyme E2L 6 (Ube2l6).
S3-12	**3.2**	1.3	plasma membrane associated protein, S3-12 (S3-12).
Ccl7	**3.1**	1.3	chemokine (C-C motif) ligand 7 (Ccl7).
Usp18	**3.1**	0.5	ubiquitin specific peptidase 18 (Usp18).
Spp1	**3.1**	1.0	secreted phosphoprotein 1 (Spp1).
Cdkn1a	**3.0**	1.0	cyclin-dependent kinase inhibitor 1A (P21) (Cdkn1a).
Oasl1	**3.0**	1.0	2'-5' oligoadenylate synthetase-like 1 (Oasl1).
Timp1	**3.0**	0.5	tissue inhibitor of metalloproteinase 1
Lrg1	**3.0**	1.5	leucine-rich alpha-2-glycoprotein 1 (Lrg1).
Zfp313	**3.0**	1.7	ring finger protein 114 (Rnf114)
Mgst1	**3.0**	1.7	microsomal glutathione S-transferase 1 (Mgst1).
Ifi27	**3.0**	1.1	interferon, alpha-inducible protein 27 (Ifi27).

Wt or *Ifit2*$^{-/-}$ mice were intranasally VSV-infected with 4x10^2 pfu, and at 2 or 6 d.p.i., brain (incl. OB) RNA expression profiles were obtained by microarray. Genes are ranked by their “fold expression level in *Ifit2*$^{-/-}$ over wt at 6 d.p.i.”. Only genes with at least 3-fold higher expression level in *Ifit2*$^{-/-}$ are included. Note: The *Ifit1/ISG56* probe of the Illumina mouse Ref-8 chip is defective and therefore the gene is not included in this list.
